# Flourishing or Languishing? Predictors of Positive Mental Health in Medical Students during the COVID-19 Pandemic

**DOI:** 10.3390/ijerph192315814

**Published:** 2022-11-28

**Authors:** Carmen Concerto, Alessandro Rodolico, Valentina Lucia La Rosa, Barbara Aiello, Miriam Martinez, Sebastiano Stuto, Carmenrita Infortuna, Laura Fusar-Poli, Maria Salvina Signorelli, Elena Commodari, Fortunato Battaglia, Eugenio Aguglia

**Affiliations:** 1 Psychiatry Unit, Department of Clinical and Experimental Medicine, University of Catania, 95123 Catania, Italy; 2 Department of Educational Sciences, University of Catania, 95124 Catania, Italy; 3 Department of Biomedical and Dental Sciences, Morphological and Functional Images, University of Messina, 98121 Messina, Italy; 4 Department of Medical Sciences, Neurology, Hackensack Meridian School of Medicine, Nutley, NJ 07110, USA

**Keywords:** flourishing, languishing, positive mental health, medical students, depression, stress, anxiety, temperament, COVID-19 pandemic

## Abstract

During the COVID-19 pandemic, medical students were burdened with high levels of stress, anxiety, and depression. The objective of the present study was to investigate predictors of positive mental health among medical students during the COVID-19 pandemic. We conducted an online survey from September 2021 to March 2022. We applied the snowball recruitment technique involving medical students from the University of Catania, Italy. We administered, anonymously, a questionnaire about demographic characteristics, the Depression Anxiety Stress Scale (DASS-21), the Temperament Evaluation of Memphis, Pisa, Paris and San Diego Autoquestionnaire (TEMPS-A), and the short form of the Mental Health Continuum (MHC-SF). Participants showed moderate anxiety, depression, and stress levels, and more than half had positive mental health status overall. This finding was inversely related to age, depression severity, cyclothymic, and depressive temperaments. Our results showed that medical students with depressive and cyclothymic temperaments were more at risk of worsening mental health status during the pandemic. Our findings may allow for further developments about the impact of personological characteristics on students’ mental health to enable more efficient support for the most vulnerable.

## 1. Introduction

Medical education is considered a time of high stress and mental workload for many graduate students. Stress has a remarkable effect on medical students’ sleep [1,2], cortical excitability [3,4], and plasticity [5,6,7]. A number of factors including academic pressure, financial concerns, fear of not acquiring the knowledge needed for the future profession, sleep deprivation, and exposure to patients’ suffering have been identified as stressor factors that negatively influence students’ wellbeing [8,9,10,11].

The Italian standard medical curriculum is compliant with the European rules of the medical degree. It might be considered as a student-centered and competency-based model of learning with basic sciences in early years and practical and clinically oriented training in later years [12].

The COVID-19 pandemic has greatly affected medical student education [12,13,14,15]. The unprecedentedly stringent precaution measures adopted by the Italian government remarkably changed students’ academic lives. University students faced new challenges due to the psychological burden induced by the pandemic and the strict measures taken to contain its propagation [16]. In Italy, most medical schools developed online learning formats with a reduction of in-person experiences. The interruption of the academic routine in terms of activities, objectives, and social relationships negatively affected students’ mental health and well-being. The extensive use of online learning system was related to increased isolation and a feeling of inability to gain research and clinical experiences [17].

Previous studies highlighted the role of students’ mental health as individual risk factor for academic performance. For instance, students’ emotional problems have been correlated to negative effects on study progress and dropout rates. [18]. The Mental Health Continuum model, described by Corey Keyes in 2002 [19], defined positive mental health as a set of positive indicators comprising emotional or hedonic components and psychological and social well-being. The described dimensions of positive mental health among the general population, defined as flourishing, may represent a protective factor from mental illness. Indeed, individuals flourishing in life are filled with positive emotions and have good psychological and social functioning [19,20]. On the contrary, low levels of positive mental health were defined as languishing, a condition of affective stagnation, in which individuals perceive their lives as empty in the absence of a diagnosable mental health condition. A state of moderate mental health is an intermediate status of neither flourishing nor languishing [19]. Previous research highlighted the role of affective temperament traits in emotional regulation to life events and, consequently, mental well-being. For instance, temperament traits are associated with the perception of stress and health risk behavior in medical students [21,22,23].

In light of these findings, in our study we aimed to further our understanding of the factors contributing to mental health in medical students during the COVID-19 pandemic. To achieve these goals, we investigated the prevalence of positive mental health during the COVID-19 pandemic in Italian medical students, and we examined the associations between psychological co-morbidities (stress, anxiety, and depression), temperament characteristics, and positive mental health. We hypothesized a pivotal role of temperament traits and psychological co-morbidities (stress, anxiety, and depression), in determining positive mental health in medical students during the pandemic. Specifically, we hypothesized that students’ emotional reactivity (temperament traits) and mental health might predict their positive mental health.

## 2. Materials and Methods

### 2.1. Design and Participants

We conducted a cross-sectional, web-based observational study between September 2021 and March 2022. Participants were invited via a link to an Airtable Form. They were recruited using the snowball technique by direct invitation, sharing a quick response (QR) code linked to the form. The questionnaire was distributed via social media (e.g., Facebook, Instagram, and WhatsApp). The platform prevented the user from moving to the next section if the previous questions were not wholly filled out, avoiding missing data. The survey was designed to be completed in less than 5 min, estimated by piloting the questionnaire with a sample of 30 psychiatry residents working in our clinic.

Eligible individuals included medical students at the University of Catania (Italy), irrespective of age, who could read and sign the informed consent section. The sample size was calculated using the Krejcie and Morgan formula for determining sample size from a given population [24]. Specifically, considering that our population consisted of 2525 medical students, a sample of at least 335 students is representative at the 95% confidence level with a standard error of 0.05.

In the online questionnaire, participants were asked whether they had received any psychiatric clinical diagnosis; if the answer was positive, participants were prevented from progressing the questionnaire. Subjects were not rewarded for participating in the study.

The survey was composed of five sections. The first section included information about the project, the study aims, the informed consent, and the researchers’ contacts. The second section included sociodemographic questions. Finally, the last three sections included three standardized questionnaires: the Depression Anxiety Stress Scale, short version (DASS-21) [25], the Temperament Evaluation of Memphis, Pisa, Paris and San Diego Autoquestionnaire (TEMPS-A) [26], and the short form of the Mental Health Continuum (MHC-SF) [27].

All data were collected anonymously and voluntarily. All participants gave their informed consent. The study was conducted in accordance with the Declaration of Helsinki and was approved by the University of Catania Psychiatry Unit review board (n. 3/2021).

### 2.2. Instruments

The Depression Anxiety Stress Scale (DASS) is a self-rated scale that measures the severity of depression, anxiety, and stress experienced in the last week. We chose the DASS considering it to be a tool of quick compilation, a relevant element in an online survey, that would still guarantee adequate psychometric properties, comparable to other more complex tools [28] that are administered separately. Moreover, it is widely used, as it was recently found in an extensive meta-analysis on the psychopathological impact of the COVID-19 pandemic on the mental health of the general population [29]. Two versions of the instruments are available: an extended, 42-item version [30] and a short, 21-item version [31]. In the present study, we used the 21-item version. Each item corresponds to a 4-level Likert frequency scale (never, sometimes, often, or always, corresponding to the score 0, 1, 2 and 3, respectively). The author suggested cut-offs for each subscale, differentiating severity levels from average to highly severe [30]. In particular, the cut off scores of the DASS-21 scale, based on the Lovibond and Lovibond manual [30], are reported in Table 1. The short version of the tool has been translated and validated in Italian by Bottesi et al. [25]. DASS showed good internal consistency in our sample (anxiety: α = 0.84; depression: α = 0.90; stress: α = 0.86).

The Temperament Evaluation of Memphis, Pisa, Paris and San Diego Autoquestionnaire (TEMPS-A) is a self-administered scale used to measure temperamental characteristics in populations of psychiatric patients and healthy individuals [32]. The decision to use the self-administered form of the TEMPS has several reasons; first, the instrument fits perfectly into the historical continuum of Western psychopathological investigation of temperamental variations [33]; second, its semi-structured interview form (TEMPS-I) has been developed on high school and college students [33], and the TEMPS-A has also been validated on students [34,35]. Together, these reasons lead us to consider it the most suitable instrument for assessing temperamental variations in our sample. It has five subscales that investigate which affective temperament is most representative of the individual, namely, cyclothymic, dysthymic, irritable, hyperthymic, and anxious temperaments. The questionnaire requires answering yes or no to each question. The score is obtained by summing the items after dividing by subscales, converting negative responses with 1 and affirmative responses with 2. The scale has been translated and validated in Italian by Preti et al. [26]. All subscales showed good internal consistency in our sample (cyclothymic: α = 0.81; depressive: α = 0.72; irritable: α = 0.67; hyperthymic: α = 0.70; anxious: α = 0.66).

The short form of the Mental Health Continuum (MHC-SF) is a self-administered questionnaire. We administered it because it was a fast-filling instrument for measuring participants’ emotional, social, and psychological well-being [36]. It consists of 14 questions to be answered using a 6-level Likert frequency scale corresponding to a score ranging from 0 to 5 [37]. The subdomains investigated include (a) emotional well-being, as the degree of satisfaction with one’s life, (b) social well-being, based on the homonym Keyes’ model, and (c) psychological well-being. The score obtained is given by the sum of all items. The tool allows subjects to be categorized by level of mental health, including flourishing, moderately mentally healthy, and languishing [19]. MHC-SF showed good internal consistency in our sample (α = 0.90). Specifically, the scoring system for categorizing participants into languishing, flourishing, and moderately mentally healthy is based on the following algorithm: if in the past month the participant has experienced at least one of the emotional well-being items with a frequency of not more than “once or twice” and has experienced at most “once or twice” six of the eleven social well-being and psychological well-being items, he or she will be considered languishing; whereas, if the subject has experienced at least one of the EWB items with a frequency of “almost every day” and with a frequency of at least “almost every day” six of the SWB and PWB items, he or she will be considered flourishing. If these criteria are not met, the subject is considered “moderately mentally healthy”. For more details about the categorical interpretation of the instrument refer to Keyes (2002) [19]. In the present study, participants were dichotomized between those with positive mental health, to which flourishing and moderate mental health subjects belong, and those who are languishing. The instrument is available in the Italian language [27].

### 2.3. Statistical Analysis

Data analyses were conducted using the Statistical Package for the Social Sciences (SPSS) version 23.0 (IBM Corporation, Armonk, NY, USA). First, a normality test (Kolmogorov–Smirnov test) was used to determine whether sample data were drawn from a normally distributed population. Next, descriptive statistics were employed to describe the sociodemographic variables, health-risk behaviors, temperament dimensions, negative emotional states (stress, anxiety, and depression), and the prevalence of positive mental health. Correlations between continuous variables were evaluated using Spearman’s correlation coefficient. Finally, multiple logistic regression analysis was used to assess which factors were predictors of positive mental health in medical students. The percentages of positive mental health (moderate mental health and flourishing) and negative mental health (languishing) are reported as a dichotomous dependent variable included in the regression model.

## 3. Results

Descriptive sociodemographic characteristics and health-risk behaviors of the participants are reported in Table 2.

DASS showed a prevalence of severe and extremely severe levels of stress (40.6%), anxiety (87.5%), and depression (66.9%). Continuous DASS-21 scores and the TEMPS-A temperament dimensions scores are reported in Table 3.

**Figure 1:**
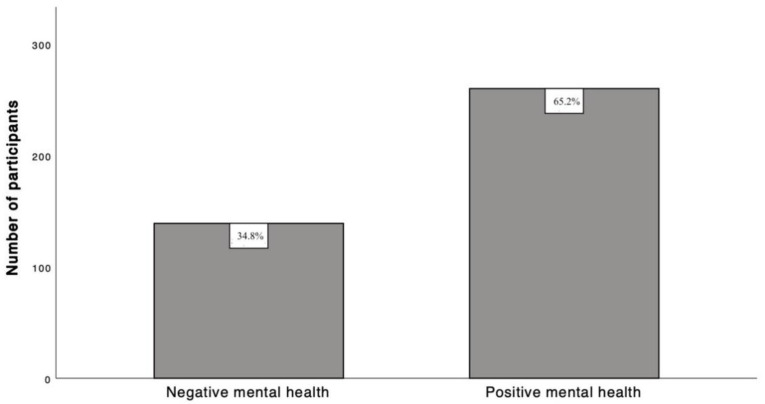
Prevalence of negative and positive mental health in the study sample.

According to the MHC-SF scale, the results indicate a moderate percentage of languishing (34.8%), a larger percentage of moderate mental health (53.4%) and a smaller percentage of flourishing (11.8%). Figure 1 describes the prevalence of positive and negative mental health state.

Statistically significant correlations were found between all the study variables. There was a statistically significant, strong positive correlation between depressive temperament and stress (r = 0.52, *p* < 0.01), depressive temperament and depression (r = 0.56, *p* < 0.01), and cyclothymic temperament and depression (r = 0.49, *p* < 0.01). Furthermore, we found a statistically significant strong negative correlation between mental health and depression (r = −0.65, *p* < 0.01) and between mental health and depressive temperament (r = −0.57, *p* < 0.01). Table 4 reports all correlations between study variables.

We finally conducted a multiple regression analysis to investigate predictors of positive mental health in medical students. A preliminary analysis suggested that the assumption of multicollinearity was met (tolerance = 0.93). The logistic regression model was statistically significant, χ^2^ (18) = 158.86, *p* < 0.0001, suggesting that it could distinguish between the students with negative and positive mental states. The model explains between 32.9% (Cox and Snell R square) and 45.5% (Nagelkerke R^2^) of the variance in the dependent variable and correctly classified 78.9% of cases. Of the predictor variables, only four were statistically significant: age, DASS depression score, cyclothymic, and depressive TEMPS-A (as shown in Table 4). Increasing age was associated with an increased likelihood of exhibiting a positive mental state, while increasing depression levels as well as cyclothymic and depressive temperament were associated with a reduction in the likelihood of exhibiting a positive mental state. Table 5 reports in detail the logistic regression results and the odd ratios and confidence intervals for the model’s predictors.

## 4. Discussion

In our study, we found that most medical students had a positive mental health status and moderate anxiety, depression, and stress levels. Furthermore, positive mental health was inversely related to age, depression severity, and cyclothymic and depressive temperaments. Indeed, although most of the sample (65.2%) fell into the positive mental health category, only 11.8% of students were flourishing, 53.4% were moderately mentally healthy, and 34.8% were languishing. Therefore, the prevalence of languishing was significantly higher than the one reported in other studies conducted on university students [38,39]. Previous studies in medical students have investigated predictors of psychological illnesses rather than the determinants of positive mental health [37]. According to Keyes’s dual-continua model [20], mental illness and positive mental health are distinct phenomena considered as part of a continuum. In keeping with this model, the participants in our study can be categorized into a positive mental health group, which is characterized by “flourishing” mental health, positive emotionality, and trusting and cooperative social relationships [40] and students with a negative mental health who are in psychological distress, show lack of interest, lack of planning, and negative emotionality, which makes them feel unfit or unprepared to face such a demanding curriculum. Therefore, the Keyes’s model supported our research hypotheses and further reinforced previous results indicating that positive mental health is associated with lack of psychological comorbidities and a better productivity [41]. The model incorporates the more traditional “hedonic” and “eudemonic” conceptualizations: a medical student with positive mental health is happy and functions very well in a demanding academic environment. At the same time, happiness and positive emotionality are in parallel with “eudaimonic” dimensions such as focus, planning, commitment to personal and academic goals, and rewarding interaction with peers [19,42]. This eudemonic perspective integrates previous theoretical models such as Ryff’s theory [42], Antonovsky’s salutogenic model [43], and the self-determination construct [44]. Considering the aforementioned literature, our results underline the importance of incorporating multiple perspectives in understanding positive and negative mental health in medical students.

A recent study by Capone et al. [23] explored positive and negative mental health in a large cohort of Italian university students during the COVID-19 pandemic and reported that 22.3% of participants were flourishing. Our results indicate a higher detrimental impact of the pandemic on medical students’ mental health. This finding is consistent with previous results showing the negative psychological impact of medical studies due to the unpredictable and uncertain nature of the pandemic event in addition to the traditionally stressful medical curriculum and the difficulties of adjusting a patient-centered curriculum to remote learning [45,46,47]. The results are in keeping with the literature [48,49,50], indicating higher psychological distress of medical students during the pandemic than the general student population [51,52]. Another interesting result concerns the relationship between temperament and medical students’ mental health. Indeed, as already reported in the literature [53,54,55], we found significant correlations between temperament and emotional state scores. Specifically, stress, anxiety and depression scores were positively correlated with anxious, depressive, cyclothymic, and irritable temperaments, confirming the study’s results by Baba et al. [53].

Furthermore, these temperaments were negatively correlated with positive mental health status. In contrast, the hyperthymic temperament, characterized by positive states such as an optimistic attitude toward life, sociality, self-assurance, creativity, resilience, and propensity to leadership [56], was negatively correlated with stress, anxiety and depression levels and positively correlated with the positive mental health. Thus, these findings confirmed temperament’s significant role in influencing medical students’ psychological well-being [53,55]. We also investigated whether having a good psychopathological compensation, i.e., belonging to the positive mental health category, was related to other variables. Results showed that age inversely correlates with positive mental health. These findings are not surprising if we consider that later-year medical students face more stressors than first-year students [46,51]. Indeed, it has been widely documented that the prevalence of stress and depression among entering medical students was low, while students engaged in medical training during the later years of the course have higher levels of anxiety, depression, and stress due to the work overload and the significant emotional impact of the relationship with patients [46,51]. Levels of depression, as well as depressive and cyclothymic temperaments, are associated with a lower likelihood of experiencing positive mental health. This result is consistent with the literature on the topic, underlining that positive mental health medical students had a lower prevalence of depression than the languishing ones [38,39]. Furthermore, depressive and cyclothymic temperaments were significantly associated with adverse psychological outcomes in medical students [53,55].

### Strengths and Limitations

This study has several strengths. First, it explored a very timely topic considering the significant impact of the COVID-19 pandemic on the mental health of medical students, which many recent studies have confirmed [45,49,50,57]. We also used a validated battery of questionnaires whose good psychometric properties were confirmed in our sample. Furthermore, the study was conducted during a period of resumption of university in-presence activities after the closures due to the COVID-19 lockdowns. As a result, the results allow us to take a snapshot of the mental health of medical students during this delicate transition from online to in-presence teaching.

However, this study also has some limitations that need to be considered. First, the study’s findings should be interpreted cautiously due to the small sample size. Furthermore, the sample was limited to medical students attending a single university, which may have had an impact on the generalizability of our results. Moreover, considering that the service used to recruit participants did not allow restricting a single user from filling out the forms, we cannot rule out with certainty that the same person was considered multiple times, although we consider this unlikely. Further studies with larger samples, including medical students from different universities, could provide more generalized data on this topic. The cross-sectional design is another limitation of this study because it does not allow for establishing an exact causal relationship between the variables investigated; longitudinal studies with large samples would be needed for this purpose. We also used a non-probability sampling method with a consequent risk of sampling bias that may affect the accuracy of the results.

## 5. Conclusions

In conclusion, our results underline the importance of incorporating multiple perspectives in understanding positive and negative mental health in medical students. Furthermore, it confirms the significant psychological impact of the COVID-19 pandemic on this group of students. In the light of the findings of this study, more attention should be paid to medical students, especially those in their final years of training, those with depressive status, and individuals with cyclothymic or depressive temperaments. In addition, our findings may allow for further developments about the impact of personological characteristics on students’ mental health to enable more efficient support for the most vulnerable, especially in the post-pandemic period.

## Figures and Tables

**Table 1:** Cut-off points for Depression Anxiety Stress Scale (DASS 21).

Severity	Depression	Anxiety	Stress
Normal	0–9	0–7	0–14
Mild	10–13	8–9	15–18
Moderate	14–20	10–14	19–25
Severe	21–27	15–19	26–33
Extremely severe	28+	20+	34+

**Table 2:** Sociodemographic characteristics and health-risk behaviors of participants (*n* = 399).

Variable		
Age		23 [21–25] ^1^
Gender	Male	279 (70.1%)
	Female	120 (29.9%)
Relationship status	Single	163 (40.9%)
	Partnered	236 (50.1%)
Academic year	1st year	36 (9%)
	2nd year	39 (9.8%)
	3rd year	50 (12.5%)
	4th year	71 (17.8%)
	5th year	57 (14.3%)
	6th year	146 (36.6%)
Psychoactive drugs	No	370 (92.7%)
	Yes	29 (7.3%)
Smoking	No	329 (82.5%)
	Yes	70 (17.5%)
Alcohol	No	165 (41.4%)
	Yes	234 (58.6%)
Coffee	No	108 (27.1%)
	Yes	291 (72.9%)
Energy Drinks	No	382 (95.7%)
	Yes	17 (4.3%)

^1^ Data are reported as median [IQR] (interquartile range).

**Table 3:** Emotional temperament dimensions, stress, anxiety, and depression of the participants.

Variable	Value ^1^
DASS stress	26 [18–32]
DASS anxiety	12 [8–20]
DASS depression	20 [14–30]
Cyclothymic (TEMPS-A)	0.58 [0.75–0.29]
Depressive (TEMPS-A)	0.44 [0.22–0.67]
Irritable (TEMPS-A)	0.13 [0–0.25]
Hyperthymic (TEMPS-A)	0.5 [0.15–0.63]
Anxious (TEMPS-A)	0.67 [0.33–1]

^1^ Data are reported as median [IQR] (interquartile range). *Note*. DASS-21 = Depression Anxiety Stress Scale 21-item, TEMPS-A = Temperament Evaluation of Memphis, Pisa, and San Diego Autoquestionnaire.

**Table 4:** Correlations between study variables.

	1.		2.		3.		4.		5.		6.		7.	8.	9.		10.		11.		12.		13.		14.		15.		16.		17.	18.
1. Age	—																															
2. Gender	0.062		—																													
3. Relationship	0.093		−0.070		—																											
4. Academic year	0.836	***	0.031		0.072		—																									
5. Drugs	0.182	***	−0.035		−0.042		0.160	**	—																							
6. Smoking	0.066		0.145	**	−0.005		0.032		0.049		—																					
7. Drinking coffee	0.067		0.004		0.136	**	0.069		−0.090		0.192	**	—																			
8. Consuming energy drink	−0.058		0.006		0.074		−0.034		0.037		0.001		0.073	—																		
9. GPA	−0.107	*	−0.164	**	0.037		0.033		−0.005		−0.001		0.027	−0.040	—																	
10. MHC–SF	−0.094		0.101	*	0.083		−0.059		−0.244	***	−0.050		0.002	−0.032	0.051		—															
11. DASS-21 Stress	0.056		−0.221	***	−0.001		0.015		0.264	***	0.079		0.036	0.033	−0.025		−0.480	**	—													
12. DASS-21 Anxiety	−0.068		−0.253	***	0.061		−0.088		0.228	***	0.056		−0.026	0.030	0.006		−0.416	**	0.661	**	—											
13. DASS-21 Depression	0.082		−0.121	*	−0.047		0.062		0.320	***	0.041		−0.039	0.031	−0.082		−0.651	**	0.696	**	0.620	**	—									
14. Cyclothymic (TEMPS-A)	−0.132	**	−0.165	**	−0.022		−0.182	**	0.068		0.157	**	−0.008	0.037	−0.132	**	−0.408	**	0.444	**	0.423	**	0.493	**	—							
15. Depressive (TEMPS-A)	0.010		−0.083		−0.057		0.010		0.161	**	0.035		0.038	0.050	−0.076		−0.570	**	0.518	**	0.423	**	0.564	**	0.401	**	—					
16. Irritable (TEMPS-A)	−0.085		0.023		0.071		−0.085		0.007		0.203	***	0.082	0.052	−0.084		−0.159	**	0.270	**	0.167	**	0.145	**	0.243	**	0.297	**	—			
17. Hyperthymic (TEMPS-A)	−0.039		0.105	*	−0.015		−0.093		−0.027		0.146	**	−0.042	0.022	−0.095		0.275	**	−0.069		−0.115	*	−0.242	**	0.062		−0.204	**	0.146	**	—	
18. Anxious (TEMPS-A)	−0.135	**	−0.249	**	0.145	**	−0.156	**	−0.002		−0.004		−0.065	−0.004	0.005		−0.183	**	0.225	**	0.300	**	0.187	**	0.326	**	0.236	**	0.079		0.000	—

*Note*. *n* = 399, DASS-21 = Depression Anxiety Stress Scale 21-item, TEMPS-A = Temperament Evaluation of Memphis, Pisa, and San Diego Autoquestionnaire, MHC-SF = Mental Health Continuum–Short Form. * *p* < 0.05; ** *p <* 0.01, *** *p* < 0.001.

**Table 5:** Predictors of positive mental health in the study sample.

	B	S.E.	*p*	OR	95% CI OR	
					Lower	Upper
Age	−0.14	0.05	0.009	0.867	0.78	0.96
Gender (Female)	0.20	0.32	0.53	1.218	0.65	2.27
Relationship (Partnered)	−0.44	0.29	0.12	0.641	0.37	1.12
Academic year	0.10	0.12	0.38	1.107	0.88	1.39
Takes drugs (Yes)	0.24	0.54	0.66	1.27	0.44	3.63
Smoke (Yes)	−0.24	0.39	0.54	0.789	0.37	1.69
Drink alcohol (Yes)	−0.02	0.29	0.94	0.978	0.56	1.72
Drink coffee (Yes)	0.05	0.32	0.88	1.05	0.56	1.97
Consume ED (Yes)	0.30	0.62	0.62	1.356	0.40	4.60
GPA	−0.01	0.02	0.62	0.988	0.94	1.04
DASS-21 Stress	0.004	0.02	0.87	1.004	0.96	1.05
DASS-21 Anxiety	0.003	0.02	0.91	1.003	0.96	1.05
DASS-21 Depression	−0.08	0.02	<0.001	0.925	0.89	0.96
TEMPS-A Cyclothymic	−1.36	0.62	0.03	0.256	0.08	0.86
TEMPS-A Depressive	−2.19	0.58	<0.001	0.112	0.04	0.35
TEMPS-A Irritable	0.12	0.72	0.87	1.123	0.27	4.60
TEMPS-A Hyperthymic	0.93	0.54	0.08	2.547	0.88	7.36
TEMPS-A Anxious	−0.03	0.41	0.94	0.972	0.44	2.16
Constant	6.74	1.72	0	847.293		

*Note.* ED = energy drinks; GPA = grade point average; DASS-21 = Depression Anxiety Stress Scale 21-item, TEMPS-A = Temperament Evaluation of Memphis, Pisa, and San Diego Autoquestionnaire.

## Data Availability

The data presented in this study are available on request from the corresponding author.
